# Testing a mindfulness meditation mobile app for the treatment of sleep-related symptoms in adults with sleep disturbance: A randomized controlled trial

**DOI:** 10.1371/journal.pone.0244717

**Published:** 2021-01-07

**Authors:** Jennifer L. Huberty, Jeni Green, Megan E. Puzia, Linda Larkey, Breanne Laird, Ana-Maria Vranceanu, Robert Vlisides-Henry, Michael R. Irwin

**Affiliations:** 1 College of Health Solutions, Arizona State University, Phoenix, Arizona, United States of America; 2 Behavioral Research and Analytics, LLC, Salt Lake City, Utah, United States of America; 3 Edson College of Nursing and Health Innovation, Arizona State University, Phoenix, Arizona, United States of America; 4 Integrated Brain Health Clinical and Research Program, Department of Psychiatry, Massachusetts General Hospital/Harvard Medical School, Boston, Massachusetts, United States of America; 5 Department of Psychology, University of Utah, Salt Lake City, Utah, United States of America; 6 Cousins Center for Psychoneuroimmunology and Mindful Awareness Research Center, Jane and Terry Semel Insitute for Neuroscience and Human Behavior, at UCLA, Los Angeles, California, United States of America; 7 Department of Psychiatry and Biobehavioral Sciences, David Geffen School of Medicine at University of California, Los Angeles, California, United States of America; Weill Cornell Medical College in Qatar, QATAR

## Abstract

The objective of this randomized controlled trial was to test whether a commercially available, mindfulness meditation mobile app, (i.e., Calm app), was effective in reducing fatigue (primary outcome), pre-sleep arousal, and daytime sleepiness (secondary outcomes) in adults with sleep disturbance (Insomnia Severity Index Score >10) as compared to a wait-list control group. Associations between the use of the Calm app (i.e., adherence to the intervention) and changes in sleep quality was also explored in the intervention group only. Adults with sleep disturbance were recruited (N = 640). Eligible and consenting participants (N = 263) were randomly assigned to the intervention (n = 124) or a wait-list control (n = 139) group. Intervention participants were asked to meditate using the Calm app ≥10 minutes/day for eight weeks. Fatigue, daytime sleepiness, and pre-sleep arousal were assessed at baseline, mid- (4-weeks) and post-intervention (8-weeks) in both groups, whereas sleep quality was evaluated only in the intervention group. Findings from intent-to-treat analyses suggest the use of the Calm app for eight weeks significantly decreased daytime fatigue (p = .018) as well as daytime sleepiness (p = .003) and cognitive (p = .005) and somatic (p < .001) pre-sleep arousal as compared to the wait-list control group. Within the intervention group, use of the Calm app was associated with improvements in sleep quality (p < .001). This randomized controlled trial demonstrates that the Calm app can be used to treat fatigue, daytime sleepiness, and pre-sleep arousal in adults with sleep disturbance. Given that the Calm app is affordable and widely accessible, these data have implications for community level dissemination of a mobile app to improve sleep-related symptoms associated with sleep disturbance.

**Trial registration**: ClinicalTrials.gov NCT04045275.

## Introduction

More than 50–70 million adults suffer from a sleep disorder (e.g., insomnia or sleep apnea)[1, 2] and 60% of Americans report significant sleep disturbances (e.g., trouble falling or staying asleep, sleeping excessively, disturbed sleep-wake schedules, restless or insufficient sleep) [3–5]. Short-term effects of sleep disturbance are associated with decreased performance (e.g., job productivity, school performance), fatigue and daytime sleepiness, while longer-term effects may have more detrimental consequences such as premature mortality, risk of cardiovascular disease, obesity, diabetes, and psychiatric disorders (e.g., anxiety, depression) [1, 6, 7]. Those with sleep disturbance, particularly with insufficient sleep, are more likely to report being obese, physically inactive, and current smokers [8] and has been declared a major public health problem [1]. Additionally, up to 10% of adults reporting sleep disturbances have severe and chronic symptoms that meet diagnostic criteria for insomnia [9], with fatigue being one of the most frequent complaints [10]. There is an urgent need to identify strategies, which can be delivered broadly at the community level, to address sleep disturbance and improve health outcomes.

Effective treatment for sleep disturbance (depending on type and severity) include both pharmacological and non-pharmacological strategies, although cognitive behavioral therapy for insomnia (CBT-I) is recommended as the first line of treatment for insomnia by the American Academy of Sleep Medicine [11, 12]. Indeed, whereas sleep medications are effective in the short-term management of insomnia, the efficacy of pharmacologic treatment is not durable due to intolerance and use is further complicated by side effects of fatigue and cognitive changes. In contrast, CBT-I is effective and durable, yet has not been widely disseminated because this treatment is intensive, poorly tolerated, costly, and/or has limited accessibility due to the need for specialized providers [13], travel, or cost of services. Novel treatments that effectively and conveniently address sleep disturbance need further investigation.

Mindfulness meditation, a less intensive intervention, has been found to be effective in the treatment of sleep disturbance, with evidence of non-inferiority to the gold standard in CBT-I [14, 15]. Such treatments are especially attractive to address sleep disturbance as they appear to reduce insomnia-related symptoms of anxiety and depression, are relatively low cost, and more accessible than clinician-administered treatment [16,^,^^17^]. A 2019 systematic review and meta-analysis of randomized controlled trials examined the effect of mindfulness meditation on sleep quality in 18 trials [18]. The findings suggest mindfulness significantly improves sleep quality (moderate strength of evidence) when compared to non-specific controls (e.g., time- and attention-matched) and may have similar effects on sleep quality as compared to evidence-based sleep treatments (e.g., CBT, sleep hygiene therapy). These findings were also durable at five- and 12-month follow-up periods, suggesting long-term benefits of mindfulness meditation on sleep quality and that mindfulness meditation is non-inferior to CBT-I. However, other systematic reviews and meta-analyses examining the effect of mindfulness meditation on sleep have been mixed with some concluding no effect of mindfulness meditation and others concluding moderate positive effects in favor of mindfulness meditation [19–23]. It is clear there is a need for more studies examining the effect of mindfulness meditation on sleep outcomes.

As the field of medicine moves towards digital health, especially in the midst of the COVID-19 pandemic, research on the delivery and efficacy of health-supportive interventions via mobile technologies such as smartphones (i.e., mHealth) is needed. Approximately 81% of US adults own a smartphone and the high utilization of this technology has opened up many opportunities for delivering mindfulness meditation via mobile apps [24], yet there are no studies to our knowledge that have examined whether a mobile app that delivers mindfulness meditation is effective in the treatment of sleep disturbance. Mobile apps may also be especially appealing to the consumer as they may mitigate common barriers and limitations to participation reported in face-to-face interventions, and thereby provide an opportunity to receive treatment and maintain necessary social distancing guidelines during the COVID-19 pandemic. Furthermore, travel is a common barrier to face-to-face interventions, and the use of mobile apps allows the participant to engage in the comfort and privacy of their home [25]. Using mobile apps to deliver mindfulness meditation may also address the issue of reporting meditation adherence as mobile apps can provide a more objective tracking method.

The availability of mindfulness meditation mobile apps has grown exponentially over the past decade [26] but the majority of these apps have not been adequately evaluated. As noted, no meditation mobile app has been tested in a randomized controlled trial for their effects on sleep disturbance [5, 27]. Previous feasibility studies testing mindfulness meditation mobile apps to improve stress or fatigue, have reported that participants enjoy using the apps and report perceived improvements in sleep [28]. Interestingly, one large cross-sectional study reported that Calm app (i.e., commercially available mindfulness meditation mobile app) users (*N* = 11,870) typically downloaded the app to improve sleep (62.9%) and adults with sleep difficulties were more likely to notice changes in their sleep after using the app compared to those without sleep difficulties [29]. The absence of rigorous research to investigate the potential benefit of commercially available mindfulness meditation mobile apps such as the Calm app to improve sleep disturbance is striking given the prevalence of sleep disturbance, widespread use of mobile apps, and emerging data that mindfulness meditation may improve sleep.

Therefore, the objectives of this study were to determine the effects of using a commercially available mindfulness meditation app (i.e., Calm) for ≥10 mins/day for eight weeks on the primary outcome, fatigue, in those who report sleep disturbance (score >10 on the Insomnia Severity Index) as compared to a wait-list control group. We also assessed the effects of using Calm on secondary outcomes of pre-sleep arousal and daytime sleepiness. Within the intervention group, associations between using Calm and changes in sleep quality were also explored. We hypothesized that fatigue, daytime sleepiness and pre-sleep arousal would significantly decrease compared to the wait-list control group. We also hypothesized that participants using Calm would report improvements in sleep quality during the intervention period, and that more time spent using Calm would be associated with improvements in sleep quality. The data gathered from this study will be used to inform future studies using mobile meditation apps as a sleep intervention.

## Methods

### Ethics approval

This study was approved by the Institutional Review Board at Arizona State University (STUDY00010050). All participants provided electronic consent prior to participating in the study.

### Research design

This study was a randomized controlled trial assessing the effects of a mindfulness meditation mobile app on sleep outcomes in adults with elevated insomnia symptoms (trial registration: ClinicalTrials.gov NCT04045275). Assessments were conducted at baseline, mid- (four weeks) and post-intervention (eight weeks). Participants were randomly assigned to the intervention group [i.e., Calm app (≥10 minutes/day)], or a wait-list control. Those randomized to the wait-list control group received the intervention after eight weeks.

### Sample size

Based on previous studies assessing the effects of in-person meditation interventions on fatigue [30, 31], power analyses were conducted in G*Power 3.1 [32]. Assuming a moderate effect size (*d* = 0.50). Results indicated that analyses with 211 participants across three time points would yield .80 power. To account for attrition, we aimed to enroll up to 250 participants.

### Recruitment and enrollment

Participants were recruited nationally between June and September of 2019 via Internet-based strategies including social media (e.g. Facebook, Twitter, Instagram), social networking sites, email listservs and ResearchMatch.org. The study was advertised as a “mobile app intervention for physical and mental health”. Interested participants completed a brief (5–10 minutes) eligibility screener via Qualtrics (i.e., online survey database). Inclusion criteria were: 1) sleep disturbance defined by a moderate level of elevated insomnia symptoms (i.e., score >10 on the Insomnia Severity Index) [33]. Because we recruited participants from the general population, we chose the cutoff score of 10 as Morin and colleagues [33] determined it to be optimal for detecting at least subthreshold insomnia in a community sample, 2) 18 years or older, 3) could read and understand English, 4) willing to download the Calm app to their smartphone, 5) had not practiced meditation for more than 60 minutes a month in the past six months, and 6) willing to be randomized. If eligible, participants were emailed a link to a video explaining the informed consent and study procedures. If interested in participating, participants completed a quiz to assure they understood study procedures. Participants then completed an electronic informed consent delivered via Qualtrics. Once consented, participants completed baseline questionnaires and then were randomized to intervention or wait-list control group and emailed study instructions. All recruitment and enrollment procedures were conducted remotely based out of a lab at Arizona State University by a trained research coordinator.

### Randomization and blinding

To conceal allocation, randomization occurred after enrollment, sequentially, on a one-by-one basis. After a participant completed baseline assessments, the research coordinator allocated that participant to a group using an online randomization tool (random.org). Group randomization was unstratified. The research coordinator and participants were unblinded to group allocation.

### Intervention

Calm is a mindfulness meditation app that is commercially available internationally. Calm provides a number of guided experience options including general guided meditations (e.g., 10-minute Daily Calm, various individual and series meditations, and sleep-specific meditations) grounded in mindfulness-based stress reduction (MBSR) and Vipassana meditation and Sleep Stories, grounded in sensory immersion and present moment awareness. Participants were asked to download the Calm app on their phone from the Apple App Store or Google Play. Participants then received a study email address and password allowing them to access their Calm account (eight-week free subscription to Calm). Participants were asked to meditate for at least 10 minutes each day and encouraged to use Calm as much as they would like during the intervention. We chose 10 minutes/day for the intervention because previous research using the Calm app suggests 10 min/day may lead to improvements in mental health [34]. The research team recommended using the “7-Days of Calm” (introduction to meditation) at the beginning of the intervention, however participants were given autonomy to choose meditations they wanted to use to replicate how a consumer might use the app (i.e., real life application). This prescription mimicked how a new, paying member would use the app (full exposure with autonomy).

### Wait-list control

Participants randomized to the wait-list control group were asked to wait eight weeks from the time they signed consent and asked not to change their normal routine or download or use any apps for relaxation, meditation or sleep. After eight weeks, participants received a Qualtrics link to the post-intervention surveys (same surveys that the intervention group received) and received free access to Calm (in the same way as the intervention group) for eight weeks upon completion of the surveys.

### Measures

Information about demographic characteristics, self-reported chronic health and sleep diagnoses was collected at baseline. During the study period, both the intervention and wait-list control groups were administered three self-reported surveys at baseline, mid- (four weeks) and post-intervention (eight weeks) that assessed fatigue, daytime sleepiness, and pre-sleep arousal. Among intervention participants only, sleep quality was assessed via daily sleep diaries throughout the study period. All data was collected using Qualtrics (online survey software).

### Primary outcome

#### Fatigue

The Fatigue Severity Scale (FSS) was used to measure fatigue [35, 36]. The FSS is a nine-item scale assessing fatigue symptoms and its effect on a person’s activities and lifestyle. The FSS utilizes a seven-point Likert scale from 1 = strongly disagree to 7 = strongly agree. Item scores are averaged to calculate a total score (range = 1 to 7). Higher scores indicate higher levels of fatigue. The FSS has high internal consistency (α = .88) and has demonstrated strong convergent validity with physiological and self-report measures in adults with sleep disturbance.

### Secondary outcomes

#### Daytime sleepiness

The Epworth Sleepiness Scale (ESS) was used to measure daytime sleepiness [37]. The ESS is an eight-item scale that asks the participants to rate how likely they are to doze off or fall asleep in while engaged in eight different activities. The questionnaire utilizes a four-point Likert scale with responses ranging from 0 = would never doze to 3 = high chance of dozing. Items are summed to produce a total score ranging from 0 to 24. Higher scores indicate higher levels of daytime sleepiness. The ESS has high internal consistency (α = .86) [38] correlates with objective measures of daytime sleepiness and physiological indicators of sleep disturbance, and can effectively distinguish between adults with and without sleep-related diagnoses [37].

#### Pre-sleep arousal

The Pre-Sleep Arousal Scale (PSAS) was used to measure pre-sleep arousal [39]. The PSAS is a 16-item scale assessing an individual’s state of arousal as they attempt to fall asleep. Eight items measure symptoms of cognitive (e.g., intrusive thoughts) arousal and eight items measure symptoms of somatic (e.g., sweating) arousal experienced at bedtime. The PSAS utilizes a five-point Likert Scale with responses ranging from 1 = not at all to 5 = extremely. Items are summed to produce a two subscale scores with totals ranging from 8 to 40. Higher scores indicate greater pre-sleep cognitive and/or somatic arousal. The cognitive and somatic subscales of the PSAS are reported to be valid and reliable measures with Cronbach’s α = 0.76 and α = 0.81, respectively [39].

#### Sleep quality

Sleep quality was measured using the gold-standard Core Consensus Daily Sleep Diary questionnaire in the intervention group only [40]. The Core Consensus Diary is associated with validated retrospective self-report measures of insomnia severity (e.g., Insomnia Severity Index) as well as with prospective objective measures (e.g., actigraphy), can effectively distinguish between good and sleepers and those with insomnia, and is sensitive to treatment-related improvements in sleep [40]. Self-monitoring or tracking has been known to act as an intervention and may impact behavior [41, 42]; therefore, we chose not to ask the wait-list control group to keep daily sleep diaries. Each day during the eight-week intervention, intervention group participants completed sleep diary entries regarding their sleep the previous night. Diaries were submitted weekly via Qualtrics. The sleep diary questionnaire contains nine questions used to gather general information about daily sleep patterns, including bedtime, nighttime awakenings, final wake time, and perceived sleep quality. Sleep quality is measured using a 1 = very poor to 5 = very good rating scale.

### Adherence to the intervention

Adherence to the intervention was tracked using data provided by Calm and confidentially shared with Arizona State University. Reports included the corresponding date and time, title, and duration of engagement for each participant.

### Incentives

Due to limited funding, monetary compensation was provided for the first 100 enrolled participants. Incentives were provided based on the percentage of sleep diaries completed: $25 for 70%, $15 for 50%, and $10 for 25%. Participants enrolled after the first 100 who completed at least 70% of their sleep diaries were entered into a drawing to win one of two $99 Amazon gift cards. All participants were provided with an additional free eight-week membership to Calm at the end of the intervention.

### Statistical analysis

Descriptive statistics (i.e., frequencies, percentages, means, *SD*s) were analyzed in IBM SPSS 26.0 to and used to characterize the sample.

Comparisons of between-group differences in changes in fatigue, daytime sleepiness, and pre-sleep arousal were assessed using repeated-measures ANOVAs. Of primary interest is the group × time interaction which, if significant, would indicate that Calm was more effective in reducing study outcomes compared to the wait-list control group. To decrease the risk of bias due to attrition, comparative models were run both as per-protocol and intent to treat (ITT) analyses [43]. Effect sizes were interpreted using Cohen’s conventional criteria, in which *d* = 0.20, *d* = 0.50, and *d* = 0.80 are understood to reflect small, medium, and large effects, respectively [44]. A *p*-value of < .05 was considered statistically significant.

Multilevel models (MLMs) were used to evaluate changes in sleep quality over time in the intervention group. Models were estimated using Hierarchical Linear and Nonlinear Modeling (HLM 8) [45], using restricted maximum likelihood estimation procedure and the default convergence criteria [46]. Cohen’s *d* was calculated by dividing the unstandardized regression coefficient by the standard deviation of the outcome variable [47].

To assess whether sleep quality improved over time, we compared a null model of weekly self-reported sleep quality to a model in which sleep quality was regressed on time (i.e., study day) at level 1.

$$SLEEP\_QUALITY_{ij}=\gamma_{00}+\gamma_{10}*TIME_{ij}+u_{0j}+r_{ij}$$

To examine the relationships between changes in sleep quality and Calm usage in the intervention group, we included Calm usage (min/week) and time × usage interaction terms as additional level-1 predictors.

$$SLEEP\_QUALITY_{ij}=\gamma_{00}+\gamma_{10}*TIME_{ij}+\gamma_{20}*USAGE_{j}+\gamma_{30}*TIME\times USAGE_{ij}+u_{0j}+r_{ij}$$

## Results

**Fig 1:**
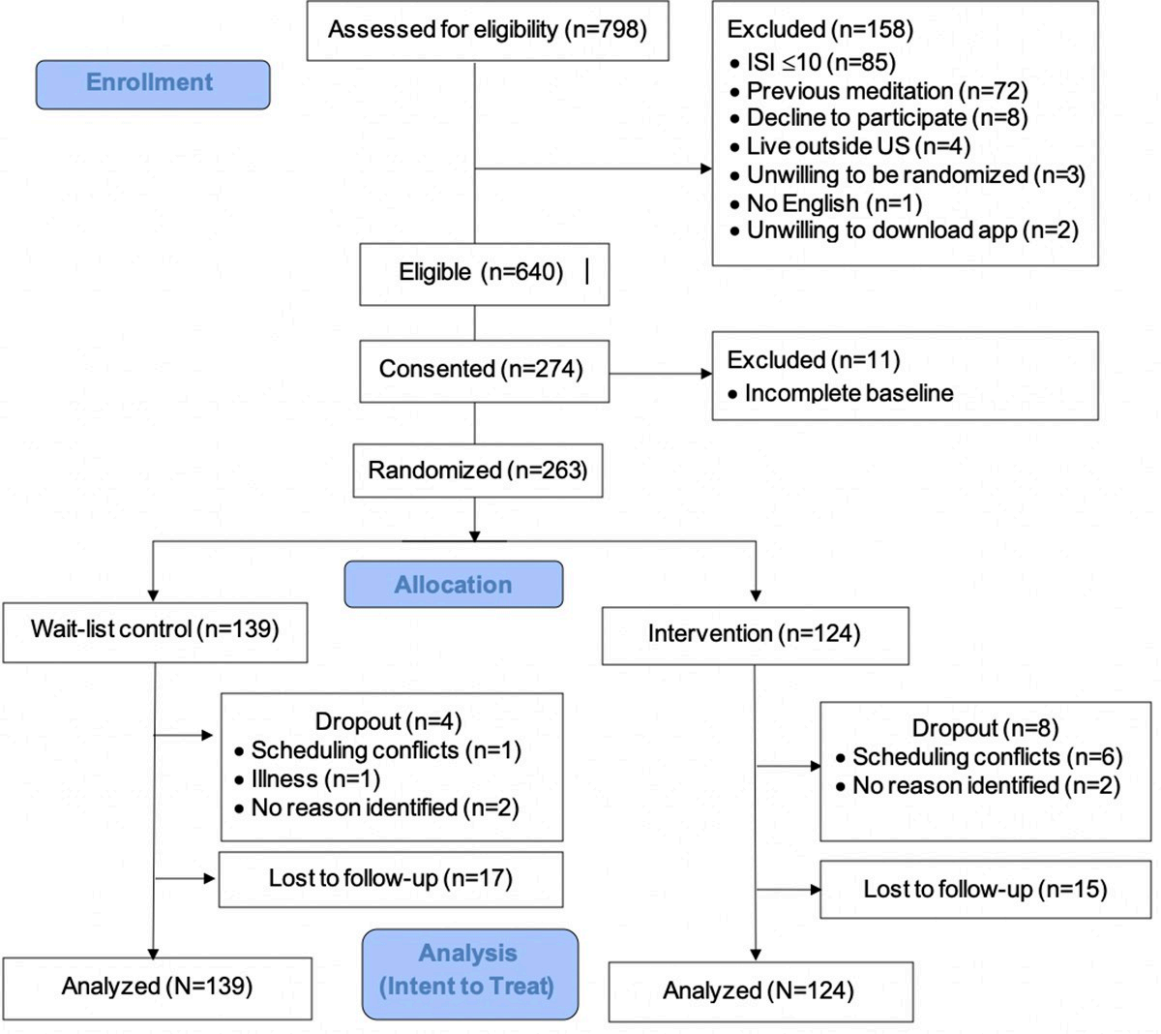
Consort diagram.

Two hundred seventy-four participants enrolled in the study; however, 11 participants were not randomized due to incomplete baseline questionnaires (Fig 1). There were 124 participants randomized to the intervention group and 139 to the wait-list control group (*N* = 263). Four participants in the wait-list control group and eight participants intervention group dropped out of the study (i.e., contacted research team to discontinue participation). There were 17 participants in the control condition and 15 in the intervention who were lost to follow-up (i.e., did not contact researchers to discontinue participation and did not complete post-study surveys). In order to ensure that definitions related to attrition were consistent across study groups, these operationalizations are based on response to study questionnaires, not on app usage or sleep diary completion. Attrition rates did not significantly differ across groups (χ^2^ = 0.56, *p* = .46). Additionally, there were no differences in age (*t* = 1.14, *p* = .26), race (χ^2^ = 2.31, *p* = .68), ethnicity (χ^2^ = 3.20, *p* = .07), gender (χ^2^ = .55, *p* = .76), income (χ^2^ = .2.38, *p* = .80), education (χ^2^ = 2.67, *p* = .75), and marital status (χ^2^ = 2.47, *p* = .78) in participants who dropped out compared to those analyzed. There were also no differences in baseline fatigue (*t* = 1.12, *p* = .24), somatic pre-sleep arousal (*t* = .40, *p* = .69), cognitive pre-sleep arousal (*t* = .29, *p* = .77), and daytime sleepiness (*t* = .45, *p* = .66) in participants who dropped out compared to those analyzed.

### Demographics and health characteristics

On average, participants were 44.50 years old (*SD* = 14.58). Our sample was racially diverse with 42.7% non-White, non-Hispanic (86.05%), and female (74.90%; see Table 1). Most participants had a college degree (70.16%), but annual household income was evenly distributed. Under half (40.55%) of participants reported being diagnosed with at least one sleep-related condition, most commonly insomnia (25.20%) and sleep apnea (15.75%).

**Table 1:** Demographic characteristics of study participants (N = 263).

Variable		N	%
Ethnicity			
	Hispanic	36	14.0
Race			
	White, European-American, or Caucasian	151	58.3
	Black or African-American	52	20.1
	Asian	34	13.1
	American Indian or Alaskan Native	7	2.7
	Other	15	5.8
Gender Identification			
	Male	56	21.6
	Female	194	74.9
	Other	9	3.5
Household Income			
	<$21,000 per year	48	18.7
	$21,000 - $40,000 per year	44	17.1
	$41,000 - $60,000 per year	48	18.7
	$61,000 - $80,000 per year	33	12.8
	$81,000 - $100,000 per year	31	12.1
	>$100,000 per year	53	20.6
Education			
	Less than high school	1	0.4
	Highschool diploma	20	7.8
	Some college	56	21.7
	Associates/2-year degree	21	8.1
	Bachelors/4-year degree	88	34.1
	Graduate school or above	72	27.9
Marital status			
	Single	81	31.3
	Partnered/In a relationship	47	18.1
	Married	98	37.8
	Separated	2	0.8
	Divorced	27	10.4
	Widowed	4	1.5
Sleep-related conditions			
	Insomnia	64	25.2
	Sleep apnea	40	15.7
	Narcolepsy	5	2.0
	Restless leg syndrome	22	8.7
	Night terrors	12	4.7
	Other	11	4.3

### Changes in study outcomes

Per-protocol and ITT analyses of the primary outcome fatigue produced similar results; as such, results described below reflect findings from ITT analyses.

#### Fatigue (primary outcome)

Means for FSS scores over time are presented in Table 2. There was no main effect of group (*F* = 0.01, *p* = .94, *d* = 0.01) There was a significant main effect of time, showing within-group decreases in fatigue in the overall sample (*F* = 4.30, *p* = .02, *d* = 0.26). The significant group × time interaction was significant (*F* = 3.30, *p* = .04, *d* = 0.23), indicating that participants using Calm had larger reductions in fatigue during the intervention period than did participants in the wait-list control group.

**Table 2:** Mean scores in primary and secondary outcomes across assessment timepoints by group.

		Mean (*SD*)							
		Intervention (*n* = 124)			Wait-list control (*n* = 139)			Value (95% CI)	
Outcome, scale range		Pre	Mid	Post	Pre	Mid	Post	Between-Groups Difference in Change Values*	*d*
Primary outcome									
	FSS, 1–7	6.1 (1.8)	5.9 (1.9)	5.8 (2.0)	6.0 (1.7)	5.8 (1.9)	6.0 (1.9)	0.2 (0.2, 0.5)	0.23
Secondary outcomes									
	ESS, 0–24	8.2 (5.1)	7.8 (4.9)	7.6 (4.5)	8.2 (4.8)	8.4 (4.8)	8.7 (4.9)	1.1 (0.8, 1.5)	0.30
	PSAS-cognitive, 8–40	25.7 (7.7)	22.7 (8.1)	22.6 (8.1)	26.1 (6.7)	25.0 (7.0)	25.0 (7.3)	2.0 (1.4, 2.7)	0.33
	PSAS-somatic, 8–40	15.2 (5.8)	13.2 (5.6)	13.1 (4.9)	15.4 (6.1)	15.2 (6.3)	15.4 (6.7)	2.6 (2.1, 3.5)	0.46

FSS = Fatigue Severity Scale; ESS = Epworth Sleepiness Scale; PSAS = Pre-sleep Arousal Scale.

*Note*. FSS scores presented above Between-group differences in change values refer to pre-post change; positive values reflect larger change among intervention group participants.

Given the diversity of the sample, exploratory analyses were used to assess whether between-group differences in improvements in fatigue were moderated by race or gender identification. Both variables were binary (White or non-White, male or female). Results showed no significant interactions between group × time interaction × race (*F* = 0.54, *p* = .58, *d* = 0.09) or group × time interaction × gender (*F* = 0.08, *p* = .92, *d* = 0.03), indicating that race status and gender do not moderate the effects of Calm on improving fatigue.

#### Secondary outcomes

Means for daytime sleepiness (ESS) and pre-sleep arousal (PSAS) scores over time are presented in Table 2. There was no main effect of group on daytime sleepiness (*F* = 0.85, *p* = .36, *d* = 0.11) or somatic pre-sleep arousal (*F* = 3.67, *p* = .06, *d* = 0.33), although main effect of group was significant for cognitive pre-sleep arousal (*F* = 4.38 *p* = .04, *d* = 0.26). There was no main effect of time on daytime sleepiness (*F* = 0.81, *p* = .44, *d* = 0.11) or somatic pre-sleep arousal. (*F* = 2.18, *p* = .12, *d* = 0.25), although the main effect for time was significant for cognitive pre-sleep arousal, with a decreased over time in the overall sample (*F* = 6.34, *p* < .001, *d* = 0.32), There were significant group × time interactions in all models of secondary outcomes including daytime sleepiness (*F* = 5.76, *p* = .003, *d* = 0.30), cognitive pre-sleep arousal (*F* = 6.90, *p* = .001, *d* = 0.33) and somatic pre-sleep arousal (*F* = 7.33, *p* = .001, *d* = 0.46), indicating that participants using Calm had larger reductions in daytime sleepiness, and cognitive and somatic pre-sleep arousal during the intervention period as compared to wait-list control group.

#### Sleep quality

Based on weekly averages of ratings in daily sleep diaries, participants using Calm reported significant improvements in perceived sleep quality. Additionally, those who spent more time using Calm (total minutes; see Adherence below) had more improvements in self-reported sleep quality (see Table 3).

**Table 3:** Changes in sleep quality in the intervention group (*n* = 124).

Model		Coefficient	*SE*	*p*	*d*
Sleep quality					
	Intercept	1.93	0.09	< .001	1.66
	Time	0.15	0.02	< .001	0.13
Sleep quality and overall Calm usage					
	Intercept	2.25	0.16	< .001	1.94
	Time	0.11	0.03	0.001	0.19
	Usage	-0.002	0.001	0.08	0.01
	Time*usage	0.0004	0.0002	0.04	0.02

Over the course of the intervention, participants using Calm also fell asleep faster (i.e., sleep latency; coefficient = -0.90, *SE* = 0.30, *p* = .02, *d* = -0.03) and slept longer (i.e., sleep duration; coefficient = 0.25, *SE* = 0.05, *p* < .001, *d* = 0.90).There were no significant changes in the frequency of nighttime awakenings (coefficient = -0.01, *SE* = 0.30, *p* = .64, *d* = 0.01).

#### Adherence to the intervention

**Fig 2:**
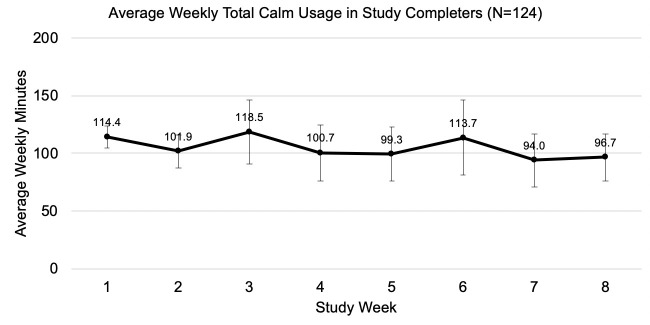
Average weekly adherence to the intervention.

Participants spent an average of 14.98 (*SD* = 26.58) minutes per day using Calm and completed 6.36 (*SD* = 10.25) sessions each week. Average weekly adherence to the intervention is presented in Fig 2. The average amount of time spent using the Calm app was relatively consistent across the intervention period (Week 1 *M* = 114.37 min, Week 8 *M* = 96.67 min), but the variability in weekly averages increased over time (Week 1 *SD* = 76.23, Week 8 *SD* = 165.67) and the percentage of participants who adhered to the intervention declined over time.

The most common time of day to use Calm was at night (i.e., 8:00 PM-4:59 AM; on average, 58.12% of sessions [*SD* = 30.31%]), followed by in the afternoon/evening (i.e., 12:00 PM-7:59 PM; *M* = 22.90%; *SD* = 22.49%) and morning (i.e., 5:00 AM-11:59 AM; *M* = 18.97%; *SD* = 19.39%). General meditations (i.e., not specifically designed for sleep) were the most commonly used component of the app (*M* = 54.24 min/week, *SD* = 38.88), followed by Sleep Stories (*M* = 8.17 min/week, *SD* = 36.26), and sleep-specific meditations (*M* = 6.61 min/week, *SD* = 25.10).

## Discussion

There is a dearth of literature related to the use of mindfulness meditation mobile apps and their effect on insomnia related outcomes. High quality studies are necessary to further understand the impact of mindfulness meditation particularly on sleep disturbance. Our study aimed to close this gap in the literature by examining the effects of using a mindfulness meditation app (i.e., Calm), ≥10 mins/day for eight weeks on fatigue in those with elevated insomnia symptoms as compared to a wait-list control group. We also assessed the effects of using Calm on daytime sleepiness and pre-sleep arousal and explored associations between using Calm and changes in sleep quality. Results suggest that using Calm led to decreases in fatigue, daytime sleepiness, and cognitive and somatic pre-sleep arousal. Using Calm was also associated with improvements in sleep quality.

### Fatigue (primary outcome)

Our findings suggest that those in the intervention group had significantly larger reductions in fatigue as compared to the wait-list control group. Specifically, average decreases in intervention participants’ FSS scores decreased by an average of 0.3 points (0.2 points larger than the decreases observed in wait-list control participants). Previous studies reported minimally important differences (MIDs) for improvement in fatigue to be between 0.08 and 0.4 points [48], which suggests that the improvements observed in the current study are clinically meaningful. To our knowledge, there are no studies to date that have examined the effects of a mindfulness meditation mobile app on fatigue in adults with elevated insomnia symptoms. Studies testing mindfulness-based interventions (not app-based) in chronic disease patients [49, 50] or cancer survivors [51] have demonstrated improvements in fatigue, however, the delivery of mindfulness meditation in most of these studies were face-to-face. In a study testing the same app as tested in this study, in cancer patients, data suggested significant decreases in self-reported fatigue. In open-ended interviews with those cancer patients reporting fatigue, 23% believed that improvements in their fatigue was a result of the meditation due possibly to sleeping better (less tired in daytime) or that the meditations allowed them a brief rest to recharge their energy levels [52, 53]. Though the mechanisms of mindfulness meditation on sleep have not yet been determined, fatigue is a common complaint in those with sleep disturbance and should be investigated in future trials.

### Secondary outcomes

The findings in this study also show promise in reducing daytime sleepiness, and cognitive and somatic pre-sleep arousal in the intervention group at eight weeks when compared to the wait-list control group. Similar to the literature in fatigue, there is limited data related to mindfulness meditation mobile app use and its effects on daytime sleepiness or pre-sleep arousal in adults with elevated insomnia symptoms. A review by Garland and colleagues (2016) suggests that mindfulness-based interventions may be particularly useful for cognitive deactivation and physiological de-arousal by allowing the individual to disengage from daily concerns and strivings and ultimately encouraging a shifting of ones relationship to sleep-related thoughts (e.g., letting go, acceptance, non-striving) [54, 55]. Our findings presented here are promising and warrant future exploration on the utility of other commercial-based meditation mobile apps on various sleep related outcomes (e.g., fatigue, daytime sleepiness, and pre-sleep arousal). In fact, two reviews on mindfulness-based interventions highlighted the need for accessible, scalable, and affordable interventions to improve sleep outcomes such as digital interventions (e.g., web- or smartphone-based) that could be encouraging directions for future research [5]. However, as noted, limited data exists on the efficacy of these platforms. Thus, there is a major opportunity for researchers to investigate the effects of commercially available mindfulness meditation mobile apps on sleep outcomes and future research in this area is warranted.

Intervention group participants reported significant improvements in perceived sleep quality over the course of the intervention period and more time spent using the mindfulness meditation app was associated with greater improvements in sleep quality. These findings are similar to mobile apps delivering CBT-I (no meditation component) suggesting that mindfulness meditation apps could be a less intensive and more affordable alternative, or adjunctive treatment, to gold standard treatment for those with sleep problems [56, 57]. For example, the app used in the current study costs $59.99/year compared to a CBT-I app at $300/year [58]. Other mobile app interventions with a meditation component have produced mixed sleep quality outcomes. One study conducted in Canada tested DeStressify, a mobile app for stress that includes meditation, in a sample of University students compared to a wait-list control group on mental health outcomes with sleep quality (measured using the Pittsburgh Sleep Quality Index) as a secondary outcome [59]. No significant differences in sleep quality were reported between groups [59]. This is likely because the (1) the intervention duration was too short to impact sleep outcomes (only four weeks) or (2) the sample did not present with significant sleep problems at baseline making it difficult to detect changes in sleep quality. Rung and colleagues (In press) evaluated the feasibility of a commercial-based mindfulness meditation mobile app (i.e., Headspace) and its association with changes in various health outcomes including sleep quality in a sample of women (N = 43) [60]. Women were asked to download and use the app for 30 days, 10 minutes per day. Findings suggest that using Headspace was associated with improvements in sleep quality sleep duration, and sleep latency [60]. However, as there was no control group and it is not possible to determine if the observed changes were due to the intervention or non-specific effects such as expectation for benefit. A review by Donker and colleagues (2013) concluded that the research regarding mental health-based mobile apps and sleep disturbance was particularly weak and there is a need for future research in this area [61]. It is clear that there is a need to test mindfulness meditation mobile apps and their effects on sleep quality, especially those currently available to the public.

#### Adherence to intervention

Participants reported robust usage of the mindfulness meditation mobile app with an average of approximately 14.98 minutes/day (6.36 sessions/week). Interestingly, participants spent substantially more time using the general meditations (i.e., not specifically designed for sleep) than other sleep-specific components of the app (e.g., Sleep Stories, sleep-specific meditations). However, the most common time for to meditate was at night (made up 52% of sessions). Despite participants using general meditations rather than sleep-specific meditations, changes in sleep outcomes were observed. It would be interesting to test if the magnitude of changes would be greater (or the same) if participants were instructed to use sleep-specific meditations prior to bed. Perhaps the effects of meditation are driven via other processes (i.e., changes in mental health) rather than directly impacting sleep. Future studies testing different types of meditation (e.g., daily, sleep-related) on sleep outcomes as well as mechanisms of change is warranted.

The attrition rate in our study was 44/263 (17%), which is similar to or lower than what has been reported in other studies of app-based meditation interventions [60]. However, this is better than studies using digitally delivered CBT-I which have reported average attrition rates of 21.6 ± 16.9% or non-completion rates of 35.2 ± 19.4% [62, 63]. In our study we used objective data provided by Calm to determine adherence, providing a more accurate measure of participation in the app compared to self-report. Similar to our study, Rung and colleagues (In press) collected objective adherence data from Headspace. Participants logged onto the app an average of 36 times (*SD* 80) for an average of 24 days (*SD* 36) with a 26% attrition rate. Their attrition was higher than our study and because objective adherence was reported as number of logins and days of use, it is unclear how many minutes of meditation were actually completed. A review by Donker and colleagues (2013) suggests that app-based health interventions may have high adherence rates because of the portability and flexible usage of mobile apps [61]. Even so, future studies should aim to provide more descriptive details regarding adherence (e.g., number of sessions completed, minutes of meditation completed, content accessed) as this information is important to further our understanding of the optimal dosage of meditation for maximal benefit. As the adherence in our study declined over time (similar to other studies), creative strategies for encouraging adherence throughout the program such as push notifications, weekly reminders, or peer support should be considered in future studies. Regardless, mobile meditation apps appear to have better attrition and completion rates than even digitally delivered CBT-I programs and should be considered as potential strategies to improve sleep in individuals with insomnia symptoms.

### Limitations

Although the findings in this study are promising, there are important limitations noted. The sample recruited was primarily female and highly educated, and therefore limits the generalizability of our findings. Although there were fairly high rates of sleep-related diagnoses in our sample (40%), it is also unclear whether the effects of the intervention would be similar in clinical settings or in populations with very severe sleep disturbance. In addition, we collected data online which may result in unrepresentative samples. Due to the remote nature of the study, we cannot determine whether or not participations were actually meditating or actively engaging with the Calm app (i.e., distracted while listening to a meditation, not paying attention, accidently left on). This is common in digitally delivered interventions [64]. Fatigue was measured as our primary outcome, but we did not require participants to report fatigue as part of our inclusion criteria. This could have attributed to the restriction of range from pre-post scores and future studies should consider adding a fatigue assessment as part of inclusion criteria. Sleep quality was not comprehensively assessed as we only analyzed one question on the sleep diaries, which provided information on subjective sleep quality, but does not capture other components which may provide a more complete representation of sleep quality (e.g., sleep latency, sleep duration, etc.) [65]. In addition to sleep diaries, future studies should include validated and reliable surveys as well as objective methods to assess sleep quality. Self-reported data presents inherent limitations (biased results, participant burden, potential data errors, incorrect interpretation of survey items) and the addition of objective measures may be less subject to bias. Additionally, sleep quality was only measured in the intervention group and therefore the intervention group is confounded by monitoring of sleep and group differences were unable to be explored on this outcome measure. Without comparison to a control group, it is unknown whether the effects observed are due to the intervention. Although, we used a wait-list control to compare our secondary outcomes. Future mHealth studies should consider using an active app-based control group to control for non-specific effects of the intervention on sleep. Finally, due to the nature of the study, participants were not blinded to treatment condition which may affect behavior especially if randomized to the wait-list control group. However, we did ask wait-list control group participants to not use any mobile apps for relaxation, meditation, or sleep during the 8-week intervention period.

## Conclusions

This is one of the first randomized controlled trials to examine the effects of a mindfulness meditation mobile app (i.e., Calm) on sleep outcomes in adults reporting elevated insomnia symptoms. Calm is an easily accessible and cost-effective resource that may be used to help sleep disturbed adults improve their sleep. Mobile apps provide a convenient way to improve sleep disturbance as users have access 24/7 in the comfort and privacy of their homes or even on the go as they please. As Calm is widely accessible, disseminating this resource as a tool for sleep can be done easily and effectively and has the potential for large reach as it is available globally.

## Supporting information

S1 File CONSORT Checklist. CONSORT 2010 checklist of information for reporting randomized trials.
(DOC)

S2 File Approved Study Protocol. Arizona State University Institutional Review Board approved study protocol.
(DOCX)

## References

[1] Liu Y., Wheaton A. G., Chapman D. P., Cunningham T. J., Lu H., and Croft J. B., “Prevalence of healthy sleep duration among adults—United states, 2014,” *Morb*. *Mortal*. *Wkly*. *Rep*., 2016, 10.15585/mmwr.mm6506a1 26890214

[2] Foundation U. H., “America’s Health Rankings analysis of CDC, Behavioral Risk Factor Surveillance System,” 2020.

[3] Kruse J. A., “Clinical Methods: The History, Physical, and Laboratory Examinations,” *JAMA J*. *Am*. *Med*. *Assoc*., 1990, 10.1001/jama.1990.03450210108045 21250045

[4] Morin C. M. and Jarrin D. C., “Epidemiology of insomnia: Prevalence, course, risk factors, and public health burden,” *Sleep Medicine Clinics*. 2013, 10.1016/j.jsmc.2013.05.002 35659072

[5] Shallcross A. J., Visvanathan P. D., Sperber S. H., and Duberstein Z. T., “Waking up to the problem of sleep: can mindfulness help? A review of theory and evidence for the effects of mindfulness for sleep,” *Current Opinion in Psychology*. 2019, 10.1016/j.copsyc.2018.10.005 30390479 PMC6459734

[6] Ferrie J. E., Kumari M., Salo P., Singh-Manoux A., and Kivimäki M., “Sleep epidemiology-A rapidly growing field,” *International Journal of Epidemiology*. 2011, 10.1093/ije/dyr203 22158659 PMC3655374

[7] Irwin M. R., “Why Sleep Is Important for Health: A Psychoneuroimmunology Perspective,” *Annu*. *Rev*. *Psychol*., 2015, 10.1146/annurev-psych-010213-115205 25061767 PMC4961463

[8] Center for Disease Control and Prevention, “Short Sleep Duration Among US Adults,” 2017 10.14423/SMJ.0000000000000632 28376532

[9] Singareddy R. et al, “Risk factors for incident chronic insomnia: A general population prospective study,” *Sleep Med*., 2012, 10.1016/j.sleep.2011.10.033 22425576 PMC3319648

[10] Ballesio A. et al., “The effectiveness of behavioural and cognitive behavioural therapies for insomnia on depressive and fatigue symptoms: A systematic review and network meta-analysis,” *Sleep Medicine Reviews*. 2018, 10.1016/j.smrv.2017.01.006 28619248

[11] Abad V. C. and Guilleminault C., “Diagnosis and treatment of sleep disorders: A brief review for clinicians,” *Dialogues in Clinical Neuroscience*. 2003 10.31887/DCNS.2003.5.4/vabad 22033666 PMC3181779

[12] Irwin M. R., Cole J. C., and Nicassio P. M., “Comparative meta-analysis of behavioral interventions for insomnia and their efficacy in middle-aged adults and in older adults 55+ years of age,” *Heal*. *Psychol*., 2006, 10.1037/0278-6133.25.1.3 16448292

[13] Williams J., Roth A., Vatthauer K., and McCrae C. S., “Cognitive behavioral treatment of Insomnia,” *Chest*, 2013, 10.1378/chest.12-0731 23381322 PMC4694188

[14] Black D. S., O’Reilly G. A., Olmstead R., Breen E. C., and Irwin M. R., “Mindfulness Meditation and Improvement in Sleep Quality and Daytime Impairment Among Older Adults With Sleep Disturbances,” *JAMA Intern*. *Med*., vol. 175, no. 4, p. 494, 4 2015, 10.1001/jamainternmed.2014.8081 25686304 PMC4407465

[15] Irwin M. R. et al., “Tai Chi Chih compared with cognitive behavioral therapy for the Treatment of Insomnia in Survivors of Breast Cancer: A randomized, partially blinded, noninferiority trial,” *J*. *Clin*. *Oncol*., 2017, 10.1200/JCO.2016.71.0285 28489508 PMC5549450

[16] Morin C. M., LeBlanc M., Daley M., Gregoire J. P., and Mérette C., “Epidemiology of insomnia: Prevalence, self-help treatments, consultations, and determinants of help-seeking behaviors,” *Sleep Med*., 2006, 10.1016/j.sleep.2005.08.008 16459140

[17] Morgan K., Gregory P., Tomeny M., David B. M., and Gascoigne C., “Self-help treatment for insomnia symptoms associated with chronic conditions in older adults: A randomized controlled trial,” *J*. *Am*. *Geriatr*. *Soc*., 2012, 10.1111/j.1532-5415.2012.04175.x 23035962

[18] Rusch H. L. et al., “The effect of mindfulness meditation on sleep quality: a systematic review and meta-analysis of randomized controlled trials,” *Annals of the New York Academy of Sciences*. 2019, 10.1111/nyas.13996 30575050 PMC6557693

[19] Kanen J., Nazir R., Sedky K., and Pradhan B., “The Effects of Mindfulness-Based Interventions on Sleep Disturbance: A Meta-Analysis,” *Adolesc*. *Psychiatry (Hilversum)*., 2015, 10.2174/2210676605666150311222928

[20] Goyal M. et al., “Meditation Programs for Psychological Stress and Well-being,” *JAMA Intern*. *Med*., 2014, 10.1001/jamainternmed.2013.13018 24395196 PMC4142584

[21] Winbush N. Y., Gross C. R., and Kreitzer M. J., “The Effects of Mindfulness-Based Stress Reduction on Sleep Disturbance: A Systematic Review,” *Explor*. *J*. *Sci*. *Heal*., 2007, 10.1016/j.explore.2007.08.003 18005910

[22] Gong H. et al., “Mindfulness meditation for insomnia: A meta-analysis of randomized controlled trials,” *J*. *Psychosom*. *Res*., vol. 89, pp. 1–6, 10 2016, 10.1016/j.jpsychores.2016.07.016 27663102

[23] Wang X., Li P., Pan C., Dai L., Wu Y., and Deng Y., “The Effect of Mind-Body Therapies on Insomnia: A Systematic Review and Meta-Analysis,” *Evidence-based Complementary and Alternative Medicine*. 2019, 10.1155/2019/9359807 30894878 PMC6393899

[24] Pew Research Center, “Mobile Fact Sheet,” 2019.

[25] Tomlinson M., Rotheram-Borus M. J., Swartz L., and Tsai A. C., “Scaling up mHealth: where is the evidence?,” *PLoS Med*., vol. 10, no. 2, 2013 10.1371/journal.pmed.1001382 23424286 PMC3570540

[26] Mani M., Kavanagh D. J., Hides L., and Stoyanov S. R., “Review and Evaluation of Mindfulness-Based iPhone Apps,” *JMIR mHealth uHealth*, 2015, 10.2196/mhealth.4328 26290327 PMC4705029

[27] Ong J. C. and Moore C., “What do we really know about mindfulness and sleep health?,” *Current Opinion in Psychology*. 2020, 10.1016/j.copsyc.2019.08.020 31539830

[28] Eckert R., Larkey L., Joeman L., and Mesa R., “Myeloproliferative neoplasm patients experience of using a consumer-based mobile meditation app to improve fatigue: Informing future directions Type of Paper: Original Paper Jennifer Huberty, PhD* College of Health Solutions,” 2019. 10.2196/14292 PMC6681641 31333197

[29] Huberty J., Vranceanu A. M., Carney C., Breus M., Gordon M., and Puzia M. E., “Characteristics and usage patterns in a convenience sample of paid subscribers to calm meditation app: Cross-sectional survey,” *JMIR mHealth and uHealth*. 2019, 10.2196/15648 31682582 PMC6858610

[30] Norman J., Fu M., Ekman I., Björck L., and Falk K., “Effects of a mindfulness-based intervention on symptoms and signs in chronic heart failure: A feasibility study,” *Eur*. *J*. *Cardiovasc*. *Nurs*., 2018, 10.1177/1474515117715843 28639841 PMC5751854

[31] Espie C. A. et al., “Effect of Digital Cognitive Behavioral Therapy for Insomnia on Health, Psychological Well-being, and Sleep-Related Quality of Life: A Randomized Clinical Trial,” 2019, 10.1001/jamapsychiatry.2018.2745 30264137 PMC6583463

[32] Faul F., Erdfelder E., Lang A. G., and Buchner A., “G*Power 3: A flexible statistical power analysis program for the social, behavioral, and biomedical sciences,” 2007, 10.3758/bf03193146 17695343

[33] Morin C. M., Belleville G., Bélanger L., and Ivers H., “The insomnia severity index: Psychometric indicators to detect insomnia cases and evaluate treatment response,” *Sleep*, 2011, 10.1093/sleep/34.5.601 21532953 PMC3079939

[34] Huberty J., Green J., Glissmann C., Larkey L., Puzia M., and Lee C., “Efficacy of the mindfulness meditation mobile app ‘calm’ to reduce stress among college students: Randomized controlled trial,” *J*. *Med*. *Internet Res*., 2019, 10.2196/14273 31237569 PMC6614998

[35] Krupp L. B., Jandorf L., Coyle P. K., and Mendelson W. B., “Sleep disturbance in chronic fatigue syndrome,” *J*. *Psychosom*. *Res*., vol. 37, no. 4, pp. 325–331, 1993 10.1016/0022-3999(93)90134-2 8510058

[36] Krupp L. B., “The Fatigue Severity Scale,” *Arch*. *Neurol*., 1989, 10.1001/archneur.1989.00520460115022 2803071

[37] Johns M. W., “A new method for measuring daytime sleepiness: The Epworth sleepiness scale,” *Sleep*, 1991, 10.1093/sleep/14.6.540 1798888

[38] Kendzerska T. B., Smith P. M., Brignardello-Petersen R., Leung R. S., and Tomlinson G. A., “Evaluation of the measurement properties of the Epworth sleepiness scale: A systematic review,” *Sleep Medicine Reviews*. 2014, 10.1016/j.smrv.2013.08.002 24135493

[39] Nicassio P. M., Mendlowitz D. R., Fussell J. J., and Petras L., “The phenomenology of the pre-sleep state: the development of the pre-sleep arousal scale,” *Behav*. *Res*. *Ther*., vol. 23, no. 3, pp. 263–271, 1985 10.1016/0005-7967(85)90004-x 4004706

[40] Maich K. H. G., Lachowski A. M., and Carney C. E., “Psychometric Properties of the Consensus Sleep Diary in Those With Insomnia Disorder,” *Behav*. *Sleep Med*., 2018, 10.1080/15402002.2016.1173556 27231885

[41] Chen J. A., Fearey E., and Smith R. E., “‘that Which Is Measured Improves’: A Theoretical and Empirical Review of Self-monitoring in Self-management and Adaptive Behavior Change,” *J*. *Behav*. *Ther*. *Ment*. *Heal*., vol. 1, no. 4, p. 19, 2017.

[42] Mairs L. and Mullan B., “Self-Monitoring vs. Implementation Intentions: a Comparison of Behaviour Change Techniques to Improve Sleep Hygiene and Sleep Outcomes in Students,” *Int*. *J*. *Behav*. *Med*., 2015, 10.1007/s12529-015-9467-1 25673110

[43] McCoy C. E., “Understanding the intention-to-treat principle in randomized controlled trials,” *Western Journal of Emergency Medicine*. 2017, 10.5811/westjem.2017.8.35985 29085540 PMC5654877

[44] Cohen J., “Statistical power analysis for the behavioural sciences. Hillside,” *NJ*: *Lawrence Earlbaum Associates*. 1988, 10.1111/1467-8721.ep10768783

[45] Raudenbush S. W., Bryk A., Cheong Y. F., and Congdon R., “HLM 8 for Windows.” Scientific Software International, Inc., Skokie, IL, 2019.

[46] Raudenbush S. W., Bryk A. S., and Congdon R., “HLM for windows (version 7),” *Comput*. *software*, *Lincolnwood*, *Sci*. *Softw*. *Int*., 2000.

[47] Feingold A., “Effect Sizes for Growth-Modeling Analysis for Controlled Clinical Trials in the Same Metric as for Classical Analysis,” *Psychol*. *Methods*, 2009, 10.1037/a0014699 19271847 PMC2712654

[48] Nordin Å., Taft C., Lundgren-Nilsson Å., and Dencker A., “Minimal important differences for fatigue patient reported outcome measures—A systematic review,” *BMC Medical Research Methodology*. 2016, 10.1186/s12874-016-0167-6 27387456 PMC4937582

[49] Grossman P. et al., “MS quality of life, depression, and fatigue improve after mindfulness training: A randomized trial,” *Neurology*, 2010, 10.1212/WNL.0b013e3181f4d80d 20876468 PMC3463050

[50] Surawy C., Roberts J., and Silver A., “The effect of mindfulness training on mood and measures of fatigue, activity, and quality of life in patients with chronic fatigue syndrome on a hospital waiting list: A series of exploratory studies,” *Behav*. *Cogn*. *Psychother*., 2005, 10.1017/S135246580400181X

[51] Carlson L. E. and Garland S. N., “Impact of Mindfulness-Based Stress Reduction (MBSR) on sleep, mood, stress and fatigue symptoms in cancer outpatients,” *Int*. *J*. *Behav*. *Med*., 2005, 10.1207/s15327558ijbm1204_9 16262547

[52] Huberty J. et al., “Smartphone-based meditation for myeloproliferative neoplasm patients: Feasibility study to inform future trials,” *J*. *Med*. *Internet Res*., 2019, 10.2196/12662 31033443 PMC6658299

[53] Huberty J., Eckert R., Larkey L., Joeman L., and Mesa R., “Experiences of using a consumer-based mobile meditation app to improve fatigue in myeloproliferative patients: Qualitative study,” *J*. *Med*. *Internet Res*., 2019, 10.2196/14292 31333197 PMC6681641

[54] Garland S. N., Zhou E. S., Gonzalez B. D., and Rodriguez N., “The Quest for Mindful Sleep: a Critical Synthesis of the Impact of Mindfulness-Based Interventions for Insomnia,” *Current Sleep Medicine Reports*. 2016, 10.1007/s40675-016-0050-3 28191449 PMC5300077

[55] Mrazek A. J. et al., “The future of mindfulness training is digital, and the future is now,” *Current Opinion in Psychology*. 2019, 10.1016/j.copsyc.2018.11.012 30529975

[56] Yu J. S., Kuhn E., Miller K. E., and Taylor K., “Smartphone apps for insomnia: Examining existing apps’ usability and adherence to evidence-based principles for insomnia management,” *Transl*. *Behav*. *Med*., 2019, 10.1093/tbm/iby014 30590862

[57] Shin J. C., Kim J., and Grigsby-Toussaint D., “Mobile Phone Interventions for Sleep Disorders and Sleep Quality: Systematic Review,” *JMIR mHealth uHealth*, 2017, 10.2196/mhealth.7244 28882808 PMC5608984

[58] Cowie J., Bower J. L., Gonzalez R., and Alfano C. A., “Multimedia Field Test: Digitalizing Better Sleep Using the Sleepio Program,” *Cognitive and Behavioral Practice*. 2018, 10.1016/j.cbpra.2017.09.005

[59] Lee R. A. and Jung M. E., “Evaluation of an mhealth app (destressify) on university students’ mental health: Pilot trial,” *J*. *Med*. *Internet Res*., 2018, 10.2196/mental.8324 29362209 PMC5801522

[60] Rung A. L., Oral E., Berghammer L., and Peters E. S., “Feasibility and Acceptability of a Mobile Mindfulness Meditation Intervention Among Women: Intervention Study.,” *JMIR mHealth uHealth*, 2020 10.2196/15943 32442147 PMC7298633

[61] Donker T., Petrie K., Proudfoot J., Clarke J., Birch M. R., and Christensen H., “Smartphones for smarter delivery of mental health programs: A systematic review,” *Journal of Medical Internet Research*. 2013, 10.2196/jmir.2791 24240579 PMC3841358

[62] Kuhn E. et al., “CBT-I coach: A description and clinician perceptions of a mobile app for cognitive behavioral therapy for insomnia,” *J*. *Clin*. *Sleep Med*., 2016, 10.5664/jcsm.5700 26888586 PMC4795288

[63] Soh H. L., Ho R. C., Ho C. S., and Tam W. W., “Efficacy of digital cognitive behavioural therapy for insomnia: a meta-analysis of randomised controlled trials,” *Sleep Med*., vol. 75, pp. 315–325, 2020 10.1016/j.sleep.2020.08.020 32950013

[64] Eckert R., Huberty R., Kosiorek J., Clark-Sienkiewicz H., Larkey S, Mesa L., “Remote monitoring of cancer patient participation in a 12-week online study: Challenges and directions for future research.,” *J*. *Meas*. *Phys*. *Behav*.

[65] Buysse D. J., Reynolds C. F. 3rd, Monk T. H., Berman S. R., and Kupfer D. J., “The Pittsburgh Sleep Quality Index: a new instrument for psychiatric practice and research,” *Psychiatry Res*., vol. 28, no. 2, pp. 193–213, 5 1989, 0165-1781(89)90047-4 [pii]. 10.1016/0165-1781(89)90047-4 2748771

