# Supplementary material for: Testing a mindfulness meditation mobile app for the treatment of sleep-related symptoms in adults with sleep disturbance: A randomized controlled trial
Source: PLoS One. 2021 Jan 7;16(1):e0244717. doi: 10.1371/journal.pone.0244717 (PMC7790277; doi:10.1371/journal.pone.0244717)
Supplement: S2 File — Arizona State University Institutional Review Board approved study protocol. (DOCX) [file pone.0244717.s002.docx]

| Instructions and Notes:   - Depending on the nature of what you are doing, some sections may not be applicable to your research. If so, mark as “NA”. - When you write a protocol, keep an electronic copy. You will need a copy if it is necessary to make changes. | |
| --- | --- |
| 1. **Protocol Title**   Feasibility and preliminary effects of a mobile app on sleep disturbance | |
| 1. Background and Objectives   Provide the scientific or scholarly background for, rationale for, and significance of the research based on the existing literature and how will it add to existing knowledge.   - Describe the purpose of the study. - Describe any relevant preliminary data or case studies. - Describe any past studies that are in conjunction to this study. | |
| More than 30% of the US population reports sleep disturbances, and the Centers for Disease Control and Prevention has identified insufficient sleep as a public health epidemic. Current evidence-based interventions for sleep disturbance (e.g., Cognitive Behavior Therapy for insomnia, Relaxation Therapy) are lengthy and limited due to the need for specialized providers. Pharmaco-therapy may help, however they have inherent limitations (e.g., tolerance, side effects, interactions) to their use. Novel treatments that effectively and conveniently address sleep disturbances (i.e., help individuals self-manage sleep) need further investigation.  Consumer based mobile applications (apps) may help individuals self-manage health conditions conveniently at low cost, without side effects and without the need of a trained provider. Mindfulness-based interventions (MBIs) targeting sleep and delivered via a mobile app may be an attractive, feasible and effective solution for individuals with sleep disturbances. Few, if any consumer-based mobile meditation apps that include sleep interventions have been tested for their effects on sleep. Thus, there is an unexplored opportunity to efficiently and effectively address the increasing problem of sleep disturbances.  Our guiding hypothesis is that the combined elements of a consumer-based mobile meditation app, Calm (e.g., daily mindfulness meditation, sleep stories and behavioral components), will be feasible, accepted and associated with sustained improvement in sleep among individuals who self-identify as having sleep disturbances.  Named “App of the Year” by Apple in 2017, and Apple Trend of the Year in 2018, Calm has 41 million users, 75,000 downloads per day, and over 1.5 million subscribers. Calm provides daily, 10-minute, guided meditations grounded in mindfulness-based stress reduction (MBSR) and Vipassana meditation. Uniquely, Calm also offers “Sleep Stories”, developed using techniques from Cognitive Behavioral Therapy and Relaxation Technique.  Calm is an innovative solution to reducing sleep disturbance for a number of reasons: (1) Calm is one of the only apps targeted to help users sleep (i.e., includes Sleep Stories with techniques to help people sleep) in addition to offering daily meditation. (2) Calm includes behavioral components to reinforce sustained practice (i.e., reminders, tracking of frequency (minutes/times used), feedback/patterns of use, ability to share status with others). In particular, Calm assesses how the user sleeps the morning following a Sleep Story (i.e., How did you sleep last night) and how mindful the user feels following a meditation (i.e., How mindful do you feel right now). Feedback is then provided in a line graph to show the user their trajectory of how they slept and their mindfulness over time allowing them to self-monitor and self-manage their sleeping/meditation behaviors. (3) Calm is a consumer-based app that is easy to use, inexpensive and easily accessible. (4) To date, there has been no research conducted to evaluate the effects of using Calm on sleep disturbance.  This is a randomized control feasibility study with an intervention (*Calm App)* and waitlist control.  **Aim 1**: Examine the feasibility (acceptability, demand) of using a consumer-based mindful meditation app (i.e., Calm) for >10 mins/day over eight weeks for 300 adults (>18 years of age) who self-identify as sleep disturbed (i.e., score >10 on the Insomnia Severity Index).  Acceptability is dually defined as participant satisfaction with content (i.e., daily meditations, sleep meditations, and Sleep Stories) and perceived appropriateness and usefulness of the behavioral components (i.e., reminders, self-monitoring (minutes/times used, patterns of use), share status) for self-management. **Hypothesis (H) 1**: At least 70% of participants will have 75% satisfaction of the daily meditation, sleep meditations and the Sleep Stories. **H2:** At least 70% of participants will perceive the behavioral components appropriate and useful for self-management of sleep disturbance.  Demand will be measured by (1) compliance to the >10 mins/day and sustained use over 8-weeks and (2) the comparative use of meditation, sleep meditation, and Sleep Stories (which was used most often or for longer periods). **H3**: At least 70% of participants will complete 70% of weekly prescribed minutes.  **Aim 2:** Explore the preliminary effects (i.e., trends in change, not powered for effects) of Calm on sleep disturbance (primary outcome) and emotional symptoms associated with sleep disturbance (secondary outcomes; i.e. stress, anxiety, depressive symptoms) as compared to those in a wait-list control group. **Research questions to explore:** Will Calm produce an improvement in sleep disturbance? Will Calm be associated with an improvement in emotional symptoms often associated with sleep disturbance? | |
| 1. Data Use   Describe how the data will be used. Examples include:   - Dissertation, Thesis, Undergraduate honors project - Publication/journal article, conferences/presentations - Results released to agency or organization | - Results released to participants/parents - Results released to employer or school - Other (describe) |
| Data will be used for publications and conferences/presentations. In addition, data related to the consumer-based mobile meditation app will be provided to Calm, our industry partner supplying the platform for the intervention, in a progress report. Data will be shared with Calm for internal purposes only (IP will be included, emails will not). Any publications or presentations will not include members of the Calm team and will only include the research team. | |
| 1. Inclusion and Exclusion Criteria   Describe the criteria that define who will be included or excluded in your final study sample. If you are conducting data analysis only describe what is included in the dataset you propose to use.  Indicate specifically whether you will target or exclude each of the following special populations:   - Minors (individuals who are under the age of 18) - Adults who are unable to consent - Pregnant women - Prisoners - Native Americans - Undocumented individuals | |
| Inclusion criteria are: (1) self-identify as sleep disturbed (i.e., score >10 on the Insomnia Severity Index), (2) >18 years of age, (3) English speaking, (4) willing to download the Calm app to their smartphone, and (5) have not practiced meditation more than 60 minutes a month in the past six months (6) willing to be randomized. Participants will report use of medication for sleep at baseline, mid-intervention and post-intervention and not excluded for medication. | |
| 1. Number of Participants   Indicate the total number of participants to be recruited and enrolled*:* We will conduct a trial in 300 individuals who self-identify as sleep disturbed to determine if the app is feasible and acceptable for the self-management of sleep. | |
| 1. Recruitment Methods  - Describe who will be doing the recruitment of participants. - Describe when, where, and how potential participants will be identified and recruited. - Describe and attach materials that will be used to recruit participants (attach documents or recruitment script with the application). | |
| Participants will be recruited nationally via Internet-based strategies including social media (e.g. Facebook, Twitter, Instagram), social networking sites and email listservs. Recruitment flyers will be posted on social media websites. ResearchMatch.org will be also be utilized as a recruitment tool for this protocol.  ResearchMatch.org is a national electronic, web-based recruitment tool that was created through the Clinical & Translational Science Awards Consortium in 2009 and is maintained at Vanderbilt University as an IRB-approved data repository. See attached recruitment blurbs and flyers. We will track our recruiting efforts, including how many potential participants complete our eligibility screen as a result of each method. | |
| 1. Procedures Involved   Describe all research procedures being performed, who will facilitate the procedures, and when they will be performed. Describe procedures including:   - The duration of time participants will spend in each research activity. - The period or span of time for the collection of data, and any long term follow up. - Surveys or questionnaires that will be administered (Attach all surveys, interview questions, scripts, data collection forms, and instructions for participants to the online application). - Interventions and sessions (Attach supplemental materials to the online application). - Lab procedures and tests and related instructions to participants. - Video or audio recordings of participants. - Previously collected data sets that that will be analyzed and identify the data source (Attach data use agreement(s) to the online application). | |
| Enrollment: Interested participants will complete a brief (5-10 minute) eligibility screener via Qualtrics (See Eligibility screener). **Participants will not be made know of sponsor name before randomization assignment to avoid disclosing the app that they will be using.** The research committee will follow a script to respond to interested participants who phone or email, and refer them to the eligibility screener (See Eligibility script). Eligible participants will be asked to sign an electronic informed consent prior to the start of the intervention. After informed consents have been completed, participants (N=300) will be identified. Once eligible participants have been identified, they will be emailed instructions. Ineligible participants will be notified by email.  Randomization: Participants will be randomized into either the intervention (*Calm App)* or waitlist control.  Control: The control group will be asked to wait 8-weeks to begin using Calm.  Intervention: Participants (n=150) will be asked to register for the consumer-based mobile meditation app on their phone. Participants (N=300) will then receive an email containing 8-weeks of free access to Calm. Participants (n=150) will be asked to use Calm at least 10 minutes per day and encouraged to use it as much as they would like during the intervention. This prescription mimics how a new, paying member would use the app (full exposure with autonomy).  Control: Participants (n=150) will be asked to complete all assessments over the 8-week period. They will receive free access to the app 8-weeks after they complete the baseline questionnaires (i.e., . directly following post assessments). They will also be asked to refrain from any participation in mindfulness activities for 8-weeks.  Tracking: Intervention participants (n=150) usage will be continuously tracked throughout the intervention by Calm.  Assessments: All outcomes will be measured at pre-, mid- (4-weeks) and post-intervention (8-weeks). Assessments administered are that of general demographics, Pre-Sleep Arousal Scale, Epworth Sleepiness Scale, Fatigued Severity Scale, Depression Anxiety and Stress Scale -21, and Hospital Anxiety and Depression Scale,  Acceptability is dually defined as participant satisfaction with content (i.e., daily meditations, sleep meditations, and Sleep Stories) and perceived appropriateness and usefulness of the behavioral components (i.e., reminders, self-monitoring (minutes/times used, patterns of use), share status) for self-management. Acceptability will be measured with an investigator-developed satisfaction survey.  Demand will be measured by (1) compliance to the >10 mins/day and sustained use over 8-weeks and (2) the comparative use of meditation, sleep meditation, and Sleep Stories (which was used most often or for longer periods). Demand will be measured by Calm (internally) and shared with Arizona State University.  Sleep disturbance (primary outcome) will be measured weekly using a gold standard daily sleep diary delivered via text or email (participant preference). Sleep difficulty will be measured using the Insomnia Severity Index within the Eligibility Survey; sleep fatigue will be measured using the Fatigue Severity Index Pre/Post; disturbance will be measured using the Pre-Sleep Arousal Scale Pre/Post; sleepiness will be measured using the Epworth Sleepiness Scale Pre/Post.  Emotional symptoms (secondary outcomes) will be measured using self-report; depressive symptoms and anxiety will be measured with the Depression Anxiety Stress Scale Pre/Post; and the Hospital Anxiety and Depression Scale. | |
| 1. Compensation or Credit  - Describe the amount and timing of any compensation or credit to participants. - Identify the source of the funds to compensate participants - Justify that the amount given to participants is reasonable. - If participants are receiving course credit for participating in research, alternative assignments need to be put in place to avoid coercion. | |
| The first 100 participants enrolled will receive incentives (paid for by Calm) for completion of their sleep diaries, and pre-, mid- and post-intervention assessments (ranging from $10-$25 depending on % of diaries completed). For completion of at least 70% they will receive $25; for at least 50% they will receive $15; for at least 25% they will receive $10. Participants will be provided an additional 8-week membership to Calm at the end of the intervention to compensate for their time in the study ($26.00 value). After the first 100 participants are enrolled, additional participants (n = 200) that complete 70% of their sleep diaries the survey will be entered into a drawing to win a $99 gift certificate to Amazon. Calm will be funding two, $99 Amazon gift cards for this study. There is no other funding for this survey. | |
| 1. Risk to Participants   List the reasonably foreseeable risks, discomforts, or inconveniences related to participation in the research. Consider physical, psychological, social, legal, and economic risks. | |
| There is no more than minimal risk for participation in this research study. Participation is voluntary and participants may refuse to participate or withdraw from the study at any time. Participants do not waive any legal rights by consenting to the study. If a participant chooses to withdraw from the study their data will be destroyed and will not be shared with Calm. | |
| 1. Potential Benefits to Participants   Realistically describe the potential benefits that individual participants may experience from taking part in the research. Indicate if there is no direct benefit. Do **not** include benefits to society or others. | |
| Participants may increase their ability to to self-monitor and self-manage their sleeping/meditation behaviors. The combined elements of a consumer-based mobile meditation app, Calm (e.g., daily mindfulness meditation, sleep stories and behavioral components) may help improve the sleep among individuals who self-identify as having sleep disturbances. Participants may also improve their emotional symptoms associated with sleep disturbance (i.e., stress, anxiety, depressive symptoms). | |
| 1. Privacy and Confidentiality   Describe the steps that will be taken to protect subjects’ privacy interests. “Privacy interest” refers to a person’s desire to place limits on with whom they interact or to whom they provide personal information. Click here for additional guidance on [ASU Data Storage Guidelines.](https://uto.sp10.asu.edu/sites/sec/isodocs/isodocs-asurite/Documents/Data%20Storage%20Guidelines%202012%20Final.pdf)  Describe the following measures to ensure the confidentiality of data:   - Who will have access to the data? - Where and how data will be stored (e.g. ASU secure server, ASU cloud storage, filing cabinets, etc.)? - How long the data will be stored? - Describe the steps that will be taken to secure the data during storage, use, and transmission. (e.g., training, authorization of access, password protection, encryption, physical controls, certificates of confidentiality, and separation of identifiers and data, etc.). - If applicable, how will audio or video recordings will be managed and secured. Add the duration of time these recordings will be kept. - If applicable, how will the consent, assent, and/or parental permission forms be secured. These forms should separate from the rest of the study data. Add the duration of time these forms will be kept. - If applicable, describe how data will be linked or tracked (e.g. masterlist, contact list, reproducible participant ID, randomized ID, etc.).   If your study has previously collected data sets, describe who will be responsible for data security and monitoring. | |
| The data will be accessible to the principal investigator, research technicians, and biostatistician. All data retrieved from questionnaires will be linked with study participant’s assigned ID#. A master list will contain subject ID#s and study participants’ contact information. All data obtained from participants will be kept separately from subject’s personal identifying information. This data will be kept on a password protected computer in the PI’s locked office on an ASU secure server. Identifiers will immediately be stored in a separate electronic file and in a separate file from the rest of the data. The research team will have a master list that will include participants identifying information. This master list will only be used if the research team needs to contact the participant for incomplete data or clarification of data. The master list will be kept separate from the participant’s data and will be kept on the PI’s password protected computer on an ASU secure server. The master list will be destroyed immediately after data is linked. All data and study records will be kept for at least 3 years following close of study per ASU policy. All electronic data will be removed from the computer’s hard drive. **Only de-identified data will be shared with the mobile app company.**  **Dr. Jenifer Huberty, the Principal research Investigator is an Associate Professor at ASU and also consults with Calm. The consent will include email information for participants to contact her with questions about the consulting relationship.** | |
| 1. Consent Process   Describe the process and procedures process you will use to obtain consent. Include a description of:   - Who will be responsible for consenting participants? - Where will the consent process take place? - How will consent be obtained? - If participants who do not speak English will be enrolled, describe the process to ensure that the oral and/or written information provided to those participants will be in that language. Indicate the language that will be used by those obtaining consent. Translated consent forms should be submitted after the English is approved. | |
| All informed consent documents will be administered electronically. Trained research committee that has completed CITI training will be responsible for administering consent forms. Participants will be asked to electronically sign their name at the bottom of the form.  There will be no non-English speaking participants included in this study. | |
| 1. Training   Provide the date(s) the members of the research team have completed the CITI training for human participants. This training must be taken within the last 4 years. Additional information can be found at: [Training](http://researchintegrity.asu.edu/training/humans). | |
| Jennifer Huberty – 12/18/2017  Ryan Eckert – 7/27/18  Danielle Serlin – 6/6/2018  Mariah Sullivan – 8/25/2016  Jeni Green – 9/25/2018  Breanna Laird – 9/23/2018  Abby Thompson – 12/19/17  Megan Puzia – 5/15/19 | |
|  | |
